# A new species of Megalommum Szépligeti (Hymenoptera, Braconidae, Braconinae);  a parasitoid of the pistachio longhorn beetle (Calchaenesthes pistacivora Holzschuh; Coleoptera, Cerambycidae) in Iran

**DOI:** 10.3897/zookeys.112.1753

**Published:** 2011-06-24

**Authors:** C. van Achterberg, M.R. Mehrnejad

**Affiliations:** 1 Dept. Terrestrial Zoology, NCB Naturalis, Postbus 9517, 2300 RA Leiden, The Netherlands; 2 Pistachio Research Institute, P.O. Box 77175.435, Rafsanjan, Iran

**Keywords:** Braconidae, Braconinae, *Megalommum*, *Curreia*, *Endovipio*, pistachio longhorn beetle, new species, Coleoptera, Cerambycidae, *Calchaenesthes pistacivora*, Cerambycidae, Palaearctic, Iran

## Abstract

A new species of the genus *Megalommum* Szépligeti (Hymenoptera: Braconidae: Braconinae), reared from the pistachio longhorn beetle (*Calchaenesthes pistacivora* Holzschuh; Coleoptera: Cerambycidae), is described and illustrated. The genera *Curreia* Ashmead, 1900 and *Endovipio* Turner, 1922 are new synonyms of *Megalommum* Szépligeti, 1900. Notes on the biology of *Megalommum pistacivorae* **sp. n.** and a key to the West Palaearctic and Oriental species are added. The following new combinations are given: *Megalommum xanthoceps* (Fahringer, 1928), **comb. n.,** *Megalommum jacobsoni* (Tobias, 1968), **comb. n.,** *Megalommum ayyari* (Watanabe, 1950), **comb. n.,** *Megalommum philippinense* (Baker, 1917), **comb. n.,** *Megalommum dodecanesi*(Ferrière, 1922), **comb. n.,** *Megalommum ceresense* (Turner, 1922), **comb. n**., *Megalommum inareatum* (Granger, 1949), **comb. n.,** *Megalommum antefurcale* (Szépligeti, 1915) **comb. n.** and *Megalommum tibiale* (Ashmead, 1906), **comb. n.**

## Introduction

In 1999 a conspicuous longhorn beetle (Coleoptera: Cerambycidae), was collected from pistachio trees, *Pistacia vera* Linnaeus and *Pistacia atlantica* subsp. *mutica* (Fischer & C.A. Meyer) at Sirjan (South Iran) for the first time. The beetle (Figs 7, 8, 10) proved to be undescribed and was named as *Calchaenesthes pistacivora* by Dr C. Holzschuh (Holzschuh 2003). According to Hashemi-Rad (2005) the pistachio longhorn beetle caused very severe damage to weakened pistachio trees. During April, 2007 the second author managed to rear a parasitoid from the longhorn beetle, which may play a role in the biological control of the pest. It proved to be a new species of the genus *Megalommum* Szépligeti near *jacobsoni* (Hymenoptera: Braconidae: Braconinae). The new species, *Megalommum pistacivorae*, is described below. It is the first record of a cerambycid host for the genus, and is the first record of the genus *Megalommum* from Iran.

## Material and methods

The material was partially reared from the larvae of the pistachio longhorn beetle boring in pistachio trees and partially collected at light in the wild pistachio growing areas of Sirjan (South Iran). The material is deposited in the Netherlands Centre for Biodiversity Naturalis at Leiden (RMNH).

For the recognition of the subfamily Braconinae, see van Achterberg (1990, 1993, 1997), for a key to the genera of Braconinae, see Quicke (1987), and for the terminology used in this paper, see van Achterberg (1988).

No taxonomic history is presented in this paper; for information, we refer to the Taxapad interactive catalogue (Yu et al. 2007 and later updates).

## Key to the genera Aphrastobracon Ashmead and Megalommum Szépligeti

**Table:** 

1	Scapus hardly or not protruding apico-ventrally (Fig. 71) and inner aspect of scapus normal apically, without double margin (Fig. 71); marginal cell of hind wing moderately wide subbasally (Fig. 65); first subdiscal cell of fore wing without dark patch; vein CU1b of fore wing subvertical and widened (Fig. 69), obsolescent or moderately inclivous and slender; [medio-basal area of second tergite semi-circular (Fig. 68) or triangular; dorso-lateral carinae of first metasomal tergite complete (Fig. 68)]; Oriental and Southeast Palaearctic; parasitoids of Noctuidae (living on Coccoidea: Kerridae) and Curculionidae	*Aphrastobracon* Ashmead, 1896
–	Scapus distinctly protruding apico-ventrally (Figs 33, 46, 59) and inner aspect of scapus with minute double margin apically (Figs 34, 48, but may be less developed in Afrotropical and West Palaearctic spp.); marginal cell of hind wing narrow subbasally (Figs 26, 38, 52), if intermediate then first subdiscal cell with dark patch and vein CU1b of fore wing strongly reclivous and more or less widened, triangular (Figs 45, 36) or parallel-sided (Fig. 60) or nearly so; [medio-basal area of second tergite triangular or rhombic, and often comparatively narrow (Figs 27, 39, 53), or area absent (*Megalommum inareatum* (Granger, 1949) comb. n.); vein 1-SR 0.5–0.7 times vein 1-M; specimens from New Guinea (= *Megalommum* s.s.) have no dorso-lateral carinae of the first tergite (Fig. 50), upper valve of ovipositor wider than lower valve (and distinctly flattened; Fig. 51), have often a rather short ovipositor, if rather long then ovipositor valves normal and lower valve slightly narrower; m-cu of fore wing more or less widened]; New Guinea, Oriental, Afrotropical and South Palaearctic; parasitoids of tunneling larvae of Cerambycidae and Xyloryctidae (*Pansepta* spp.)	*Megalommum* Szépligeti, (Febr.) 1900 s.l.

**Notes**. *Curreia* Ashmead, [Oct.] 1900 (Figs 26–37) and *Endovipio* Turner, 1922 (Figs 52–64) are new junior synonyms. *Endovipio* was synonymized with *Curreia* by Falco and Quicke (1997) and *Curreia* is here synonymized with *Megalommum* (syn. n.). The differences between the genera *Megalommum* Szépligeti s.s. (species from Australasia), *Endovipio* Turner (Afrotropical) and *Curreia* Ashmead (remainder of Palaeotropical area and South Palaearctic) are gradual and, therefore, they are considered congeneric here. The only small difference that seems to be valid is the development of the dorso-lateral carinae of the first tergite posteriorly; absent in *Megalommum* s.s. and present (but often only weakly developed) in *Curreia* and *Endovipio*. The shape of the ovipositor valves is variable in both groups as is the relative length of veins 1-CU1 and 1-M of the fore wing.

## Key to West Palaearctic and Oriental species of the genus Megalommum Szépligeti

**Table:** 

1	Vein CU1b of fore wing triangular, strongly widened basally (Figs 1, 2, 21, 45)	2
–	Vein CU1b of fore wing parallel-sided or slightly narrowed apically and more or less evenly widened (Fig. 36)	4
2	Medio-basal area of second metasomal tergite smooth and slightly longer than half length of tergite (Figs 2, 18, 19, 23, 24); vein CU1b of fore wing weakly reclivous (Figs 2, 22); vein 3-CU1 of fore wing 2–3 times as long as vein CU1b (Figs 2, 22); body yellowish-, orange- or dark reddish-brown; pterostigma partly dark brown (Figs 1, 2); veins more extensively dark brown (Figs 2, 21); first-fourth metasomal tergites of male largely rugulose to largely smooth (Figs 19, 24)	3
–	Medio-basal area of second metasomal tergite longitudinally rugulose and distinctly longer than half as long as tergite (Fig. 19 in Quicke et al. 2000); vein CU1b of fore wing strongly reclivous; vein 3-CU1 of fore wing about 1.2 times as long as vein CU1b; body pale yellowish; pterostigma completely yellow; veins (except dark veins below blackish parastigma) yellowish; first-fourth metasomal tergites of male largely rugose; Afrotropicalbut reportedfrom Yemen (Quicke et al. 2000)	*Megalommum xanthoceps* (Fahringer, 1928), comb. n.
3	Vein 1-M of fore wing 0.7–1.1 times as long as vein 1-CU1 (Figs 21, 77, 78); hind femur more robust (Fig. 25); hind femur dorsally and coxa yellowish-brown or brown (Fig. 25), at most hind femur dark brown dorsally; pterostigma (near vein r) dark brown (Figs 21, 77, 78); OOL of ♀ short (Fig. 22); [vein 3-SR of fore wing 1.4–1.7 times vein 2-SR; body completely dark reddish-, orange or yellowish-brown; pterostigma baso-posteriorly (and apex narrowly) yellow and remainder dark brown]; Central Asia to Cape Verde Isles, including Spain, France, Croatia, Egypt, Yemen (RMNH) and Morocco	*Megalommum jacobsoni* (Tobias, 1968), comb. n. Notes. *Curreia antefurcalis* Szépligeti, 1915 sensu Papp (1972) from Croatia belongs to *Megalommum jacobsoni* according to Falco and Quicke (1997); *Megalommum antefurcale* (Szépligeti, 1915) comb. n. is an Afrotropical species. *Megalommum jacobsoni* is closely related to *Megalommum tibiale* (Ashmead, 1906) comb. n. from China and Japan.
–	Vein 1-M of fore wing 1.4–2.2 times as long as vein 1-CU1 (Figs 2, 74, 75); hind femur less robust (Fig. 20); hind femur dorsally and coxa dark brown (Figs 1, 20), at most hind coxa largely brown; pterostigma medially (near vein r) nearly always partly yellow (Figs 2, 74, 75); OOL of ♀ slightly longer (Fig. 16); Iran	*Megalommum pistacivorae* sp. n.
4	Vein 2-SC+R of hind wing vertical; first subdiscal cell of fore wing with distinct dark spot and area below parastigma darkened; pterostigma largely yellow; marginal cell of hind wing parallel-sided submedially; vein 2–1A of fore wing nearly straight or slightly curved; Oriental	5
–	Vein 2-SC+R of hind wing longitudinal; first subdiscal cell of fore wing without distinct dark spot and area below parastigma hardly or not darkened; pterostigma partly dark brown (except of *Megalommum fasciatipenne*); marginal cell of hind wing more or less narrowed submedially; vein 2–1A of fore wing distinctly curved; [vein m-cu of fore wing narrow; scapus black]; Afrotropical and West Palaearctic	6
5	Vein 2-SR of fore wing about as long as vein r-m; vein m-cu of fore wing widened; scapus yellow with a black streak; vein CU1b of fore wing about 0.7 times as long as vein 3-CU1; = *Aphrastobracon maculipennis* Ramakrishna Ayyar, 1926, not *Megalommum maculipenne* Cameron, 1914)]; India	*Megalommum ayyari* (Watanabe, 1950), comb. n.
–	Vein 2-SR of fore wing about twice as long as vein r-m; vein m-cu of fore wing slender; scapus black; vein CU1b of fore wing about as long as vein 3-CU1; Philippines	*Megalommum philippinense* (Baker, 1917), comb. n.
6	Vein cu-a of fore wing subinterstitial and subvertical (Figs 26, 36); vein CU1b of fore wing about as long as vein 3-CU1 and subhorizontal (Fig. 36); eyes deeply incised (Fig. 28); maximum width of the subdiscal cell of fore wing about equal to width of discal cell (Fig. 26); apical half of pterostigma largely yellow; vein m-cu of fore wing subinterstitial (Fig. 26); [second metasomal suture crenulate; surroundings of medio-basal area of second tergite rugulose]; Afrotropical but reported from Egypt (Quicke et al. 2000)	*Megalommum fasciatipenne* (Ashmead, 1900), comb. n.
–	Vein cu-a of fore wing far antefurcal and reclivous (Fig. 60); vein CU1b of fore wing about half as long as vein 3-CU1 and oblique (Fig. 60); eyes shallowly incised (Fig. 54); maximum width of the subdiscal cell of fore wing about 1.4–1.7 times width of discal cell (Fig. 52); apical half of pterostigma dark brown; vein m-cu of fore wing distinctly antefurcal (Fig. 52); [second metasomal suture and surroundings of medio-basal area of second tergite smooth; only males known]; Greece and Israel	*Megalommum dodecanesi* (Ferrière, 1922), comb. n. Notes. Very close to *Megalommum ceresense* (Turner, 1922) comb. n. from South Africa and Namibia; seems to differ mainly by having the maximum width of the subdiscal cell of fore wing about 1.4 times width of discal cell (Fig. 60); about 1.6 times in *Megalommum dodecanesi*).

## Taxonomy

### Megalommum pistacivorae sp. n.

urn:lsid:zoobank.org:act:16F7B146-34DD-40BB-A3CF-CA5D7806C8A4

http://species-id.net/wiki/Megalommum_pistacivorae

**Figures 1–6.**
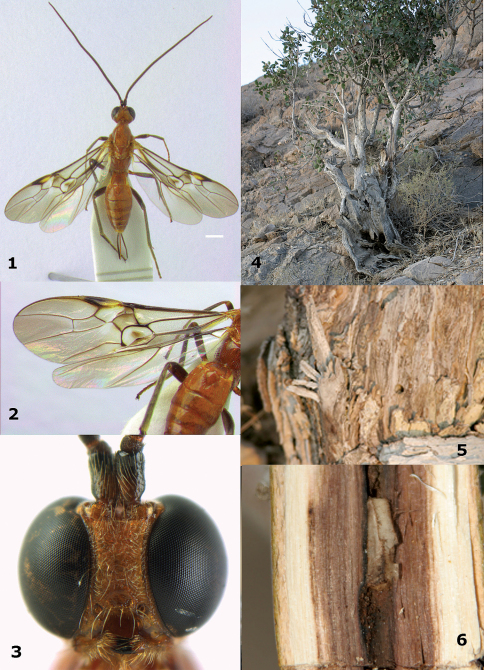
**1–3:** *Megalommum pistacivorae*sp. n., holotype female. **1** habitus dorsal (scale line = 1 mm) **2** wings and metasoma dorsal **3** head anterior. **4–6:** Old *Pistacia khinjuk* tree. **4** infested tree **5** old emergence holes of *Calchaenesthes pistacivora* **6** tunnel of *Calchaenesthes pistacivora* with closed cocoon of *Megalommum pistacivorae*.

Figs 1–3 16–20 74–76 

#### Type material.

 Holotype, ♀ (RMNH), “Iran, Sirjan, ex *Calchaenesthes pistacivora* (Ceramb.) on *Pistacia vera*, em. 14.iv.2007, M.R. Mehrnejad, RMNH’08”. Paratypes (3 ♀ + 4 ♂; RMNH): 1 ♂ topotypical and from same host, but emerged 12.xi.2007; 2 ♀ “Iran, Sirjan, 14.v.2009, at light, M.R. Mehrnejad, RMNH’09”; 1 ♀ + 1 ♂, id., but 17.v.2009; 2 ♂, id., but 30.iv.2009.

#### Diagnosis.

Body yellowish- or orange-brown; hind femur and coxa dark brown (Figs 1, 20); pterostigma medially (near vein r) partly yellow (Figs 2, 74, 75); OOL of ♀ slightly longer (Fig. 16); vein CU1b of fore wing triangular, strongly widened basally and weakly reclivous (Figs 2, 74, 75); vein 3-CU1 of fore wing 2–3 times as long as vein CU1b; vein 1-M of fore wing 1.4–2.2 times as long as vein 1-CU1 (Figs 2, 74, 75); vein 2–1A distinctly bent (Fig. 2); hind femur rather slender (Fig. 20); medio-basal area of second metasomal tergite smooth and slightly longer than half length of tergite (Figs 2, 18, 19); ovipositor sheath 0.25 times as long as fore wing and half as long as metasoma.

#### Description.

 Holotype, ♀, length of body 7.6 mm, of fore wing 7.3 mm.

*Head*. Antenna about as long as fore wing, with 61 segments; apical antennal segment with distinct spine and slender, scapus robust and distinctly protruding apically, its inner aspect with minute double margin apically; third, fourth and penultimate segments 1.2, 0.9 and 1.6 times their maximum width, respectively; length of maxillary palp 0.9 times height of head; eye distinctly emarginate (Fig. 3); face narrow and irregularly rugulose; clypeus flat, partly smooth and with some microsculpture, dorsally with angled carina and ventral margin thin and lamelliform, with 5 long setae ventrally; hypoclypeal depression 0.7 times as wide as minimum width of face (Fig. 3); frons moderately concave behind antennal sockets, smooth, without median carina (Fig. 16); vertex slightly convex, smooth, sparsely setose and stemmaticum protruding and surrounded by a groove; OOL:diameter of posterior ocellus:POL = 5:10:4; in dorsal view length of eye 5.2 times temple; temples directly narrowed behind eyes and smooth (Fig 16); malar suture absent, eye almost touching base of mandible; mandible strongly twisted and unidentate.

*Mesosoma*. Length of mesosoma 1.6 times its height; side of pronotum and propleuron smooth, except for some indistinct fine crenulae anteriorly and some punctulation posteriorly; pronotum vertical anteriorly and with a shallow groove and no antescutal depression; mesopleuron smooth, setose, except for a medial glabrous area; mesosternal sulcus smooth and narrow; metapleuron punctulate, convex; mesoscutum nearly completely sparsely punctulate and setose; notauli only present anteriorly and shallowly impressed; scutellar sulcus present and with distinct fine crenulae; scutellum weakly convex and sparsely punctulate; side of scutellum smooth; metanotum medio-anteriorly with carina, posteriorly evenly convex and smooth; propodeum smooth, setose and evenly convex.

*Wings*. Fore wing (Figs 1, 2): 1-M 1.8 times as long as 1-CU1; m-cu widened and 1.1 times as long as 1-M; first subdiscal cell with narrow and glabrous sclerome; 3-SR and SR1 weakly curved; r:3-SR:SR1 = 2:5:13; 2-SR:3-SR:r-m = 18:25:19; r-m largely unsclerotised; wide ring around dark patch of first subdiscal cell glabrous, but posteriorly setose; CU1b triangular and strongly widened basally. Hind wing: SR sinuate and marginal cell somewhat widened apically; subbasal cell setose; 1r-m weakly curved; M+CU:1-M:1r-m = 6:18:5.

*Legs*. Tarsal claws simple and with bristly setae ventrally; hind femur slender (Fig. 20) compared to *Megalommum jacobsoni*; length of femur, tibia and basitarsus of hind leg 3.9, 10.0 and 4.7 times their maximum width, respectively; hind tibia with dense adpressed setae; hind tibial spurs 0.5 and 0.6 times as long as hind basitarsus; inner side of hind tibia and tarsus with dense long whitish setae.

*Metasoma*. Length of first tergite 1.1 times its apical width, parallel-sided, dorso-lateral carinae strong behind spiracles and medial area longitudinally rugose; second tergite smooth except for crenulae near distinct triangular medio-basal area, area surrounded by crenulate groove and tergite antero-laterally with small triangular smooth areas (Fig. 18); second suture distinct and weakly sinuate and finely crenulate; medial length of second tergite 1.2 times median length of third tergite; third and following tergites smooth; ovipositor depressed, without notch or nodus dorsally and without ventral teeth, apically narrowed (Fig. 76); ovipositor straight, its sheath 0.25 times as long as fore wing.

*Colour*. Yellowish-brown; antenna, hind femur dorsally, hind tibia and tarsus and ovipositor sheath, black; fore and middle femora, tibiae and tarsi, and remainder of hind leg more or less dark brown; most veins of basal half of wing, sclerome of first subdiscal cell and apical 0.6 of pterostigma largely dark brown (Fig. 2), vein 1-R1 of fore wing yellowish and remainder of veins brown; wing membrane moderately infuscate, below parastigma and patch in first subdiscal cell dark brown.

*Variation*. Length of body of ♀ 6.7–8.7 mm (of ♂ 5.2–7.3 mm), and of fore wing of ♀ 6.7–8.1 mm (of ♂ 4.3–6.4 mm); antenna of ♀ with 54 (1), 61 (1) or 65 (1) segments, of ♂ with 44 (1), 47 (1), 51 (1) or 52 (1) segments; vein 1-M of fore wing 1.4–2.2 times as long as vein 1-CU1; vein 3-SR of fore wing 1.4–1.7 times vein 2-SR; body completely reddish-, orange or yellowish-brown; basal 0.4 of pterostigma largely yellowish or darkened and only with yellowish basal patch; hind coxa and femur largely dark brown to largely brown; length of first tergite 1.1–1.3 times its apical width; second metasomal suture weakly to strongly crenulate; second, third and base of fourth metasomal tergites of ♂ finely rugulose, but sometimes only superficially so and partly smooth; length of ovipositor sheath 0.25–0.26 times fore wing.

#### Distribution.

 South Palaearctic (Iran).

#### Biology.

 Solitary and possibly endoparasitoid of *Calchaenesthes pistacivora* Holzschuh (Coleoptera: Cerambycidae) on *Pistacia vera* Linnaeus, *Pistacia atlantica* subsp. *mutica* (Fischer & C.A. Meyer) and *Pistacia khinjuk* Stocks.

#### Etymology.

 Named after the specific name of its host.

## Biology

**Figures 7–15.**
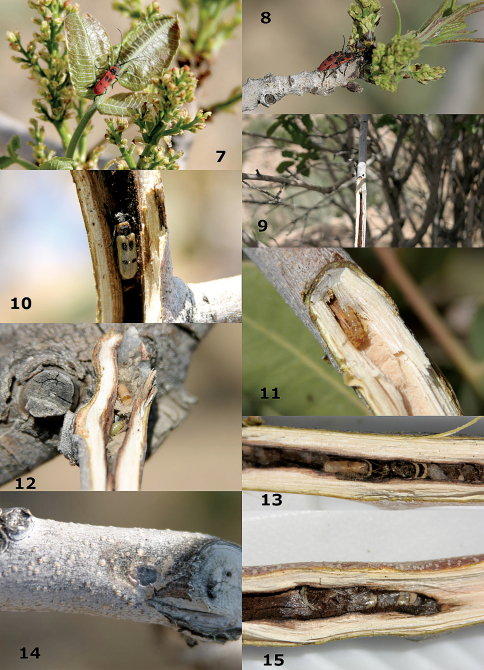
**7–11:** *Calchaenesthes pistacivora* Holzschuh. **7** adult on pistachio tree **8** copula on pistachio tree **9** tunnel and emergence hole **10** freshly emerged adult **11** larva in tunnel in spring. **12–15:** Tunnels and emergence holes in pistachio trees. **12** opened cocoon of crabronid wasp and larvae of *Hylaeus* sp. **13** cocoon of crabronid wasp **14** grey coverage of emergence hole by *Hylaeus* sp. **15** *Hylaeus* cells.

The development of the cerambycid host *Calchaenesthes pistacivora* Holzschuh lasts two years; the first winter it survives as a larva and during the second autumn it usually develops into an adult (Hashemi-Rad 2005). The adult beetle stays inside the feeding canal (Fig. 10) for about five months, from mid October to late March. In early April, the adult beetles appear on the pistachio trees and usually feed on the fresh pistachio leaves (Fig. 7); the resulting damage is considered to be minimal. The beetles lay their eggs on twigs, branches or stems of weakened pistachio trees, preferably on the pruned sites where the tiny larvae promptly penetrate into the twigs or branches. Changes in the environmental conditions of pistachio growing areas in the collection site are thought to be drought, an increase of salinity in irrigation water, which in turn is caused by a decrease of water resources and mismanagement by pistachio producers. These appear to be the major reasons for the establishment and development of this pest on cultivated pistachio trees (Hashemi-Rad 2005). It is predicted that the contaminated areas will expand as more pistachio trees lose vigor. Our survey in wild pistachio growing areas of Sirjan clearly showed that *Calchaenesthes pistacivora* had already been living in the wild pistachio trees for a long time, because the beetle canals are clearly visible on both dead and living trunks of very old trees of *Pistacia khinjuk* Stocks (Figs 4, 5) and *Pistacia atlantica mutica*. It is assumed that the beetle has been a minor phytophagous pest on wild pistachio trees for hundreds of years, however, populations increased due to weakening of these trees. The wild pistachio species have been suffering from the effects of erosion caused by human activities, particularly overgrazing and harvesting for use as firewood or charcoal as well as from severe drought periods for several years. In addition, the cultivated pistachio regions at Sirjan are not far from areas with wild pistachio trees. Therefore, emigration of *Calchaenesthes pistacivora* to pistachio orchards is likely. At present the pistachio growers experience many serious problems caused by pests and diseases (Mehrnejad 2001) and the longhorn beetle is one of those that can cause considerable tree damage and reduction of yields.

The cocoons of the parasitoid were collected inside the tunnels of the beetle (Fig. 6). The cocoons are 12–15 mm long, brownish and consist of thin paper-like silk. The beetle usually pupates inside the tunnel about 2–3 cm from the emergence hole, where the cocoons of *Megalommum pistacivorae* are also found. Field studies showed that the parasitoid enters the tunnel of the beetle through the hole made by the last instar beetle larva before pupation. The parasitoid attacks the last instar beetle larva in September and October and the parasitoid larva is solitary. Members of Braconinae are all thought to be ectoparasitoids. Surprisingly, the second author did not see any parasitoid larva or egg when he collected a fully developed beetle larva from the feeding tunnel. The beetle larva (still in the tunnel) was transferred to the laboratory where it was kept under controlled conditions. After a week a naked pupa of the wasp (that did not construct the usual cocoon) was present and the pupa developed into an adult *Megalommum pistacivorae*. Further investigations are necessary to rule out the possibility that a minute wasp larva was present on the non-exposed side of the beetle larva.

*Megalommum pistacivorae* overwinters in the prepupal stage and it has an obligatory diapause to pass the winter. The parasitoid cocoons containing the prepupa were kept under controlled conditions (27.5°C, 16L:8D photoperiod and 55±5 RH), but they did not pupate until early March. In the field, pupation takes place from mid to late March and the adults of *Megalommum pistacivorae* appear in the orchards from early April onwards. The rate of parasitism was estimated to be about 23% (n = 110) in experimental orchards. Alternative hosts for this parasitoid remain undetermined, also it is not clear if the adult wasps attack another host after emergence in early April or they pass the hot summer period and attack the larvae of *Calchaenesthes pistacivora* the following September.

In the same tunnels several other insects were found; the most common was a bee belonging to the genus *Hylaeus*. It uses the tunnels as shelter for its brood cells. Several small cells of bees are usually found at the same site where *Megalommum pistacivorae* makes its cocoons (Fig. 15). From late September till late October the female bee makes 3–5 small cells (4 mm length and 3 mm diameter each) in each tunnel, and fills them with a yellowish jelly mixed with pollen. In each cell an egg or a young larva was found (Fig. 12). The female bees cover the emergence hole (tunnel cup) with a very thin and delicate grayish waxy layer (Fig. 14). The cover protects the offspring of the bee as well as that of the wasp, *Megalommum pistacivorae*, against predation or parasitism during the overwintering period. The bee larvae overwinter in these cells at the prepupal stage and pupate from late March on. The tunnels are also used as a nest site (Fig. 13) by a Crabronid wasp.

**Figures 16–25.**
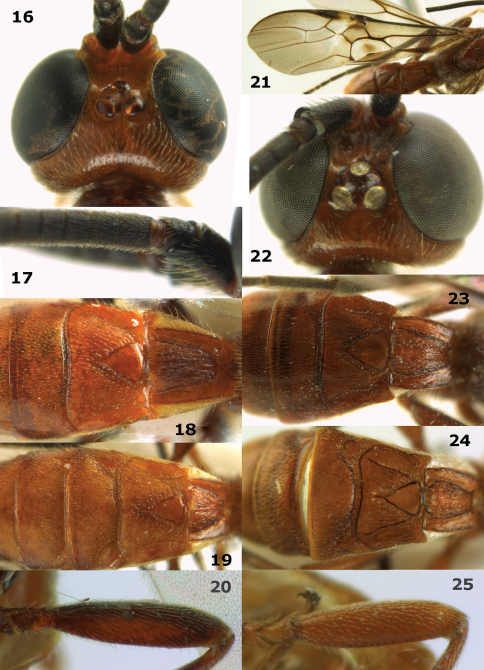
**16–20:** *Megalommum pistacivorae*sp. n., holotype female, but 19 of paratype male. **16** head dorsal **17** basal part of antenna **18, 19** basal metasomal tergites, dorsal **20** hind femur lateral. **21–25:** *Megalommum jacobsoni* **(**Tobias), Yemen, Al Kowd, female, but 24 of male. **21** fore wing **22** head dorsal **23, 24** basal metasomal tergites, dorsal **25** hind femur lateral.

**Figures 26–37.**
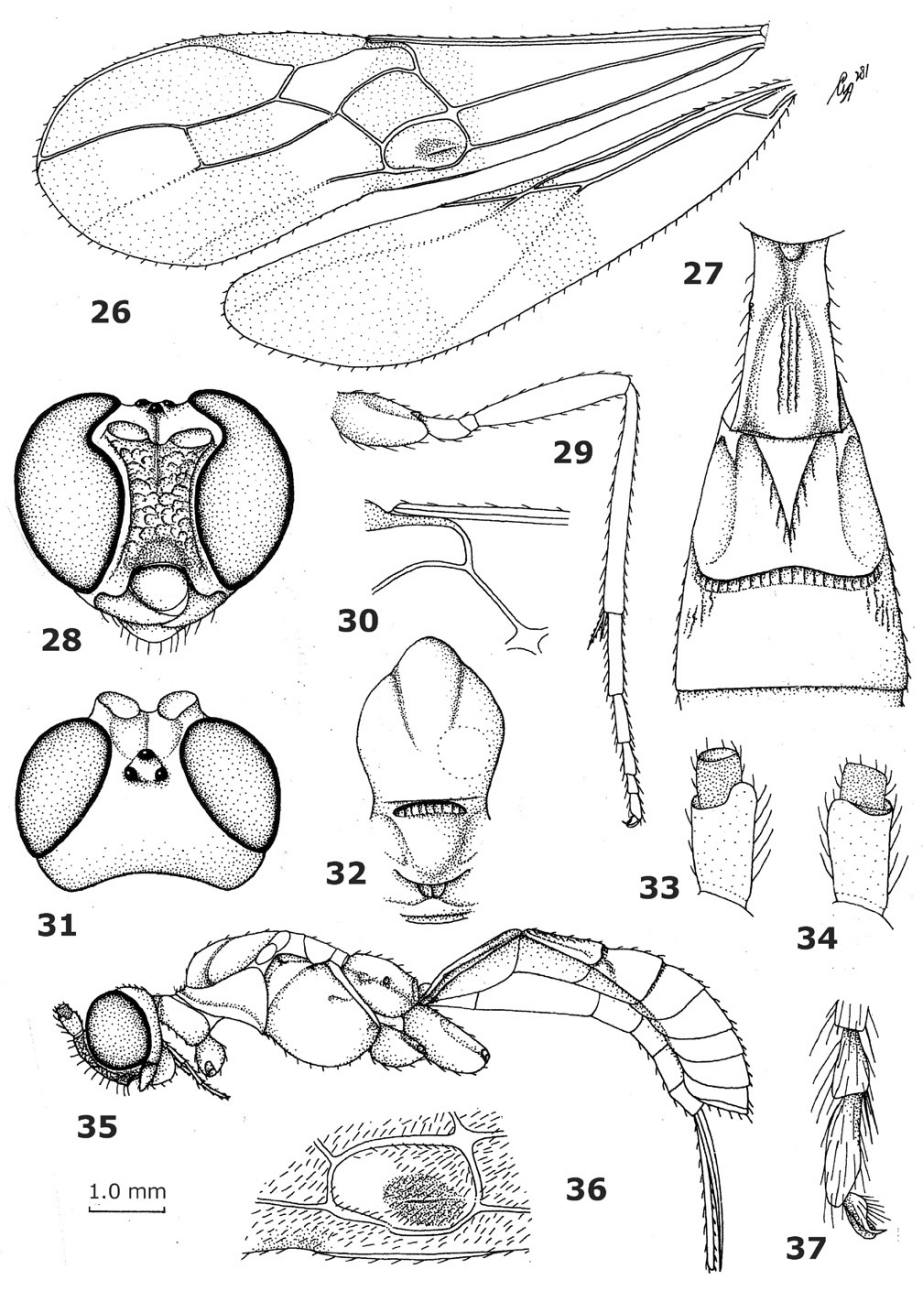
*Curreia fasciatipennis* Ashmead, holotype female. **26** wings **27** first-third metasomal tergites dorsal **28** head, anterior **29** hind leg **30** veins 1-SR and 1-M of fore wing **31** head dorsal **32** mesonotum dorsal **33** scapus and pedicellus outer side **34** scapus and pedicellus inner side **35** habitus, lateral **36** first discal and first subdiscal cells of fore wing **37** inner hind claw.

**Figures 38–51.**
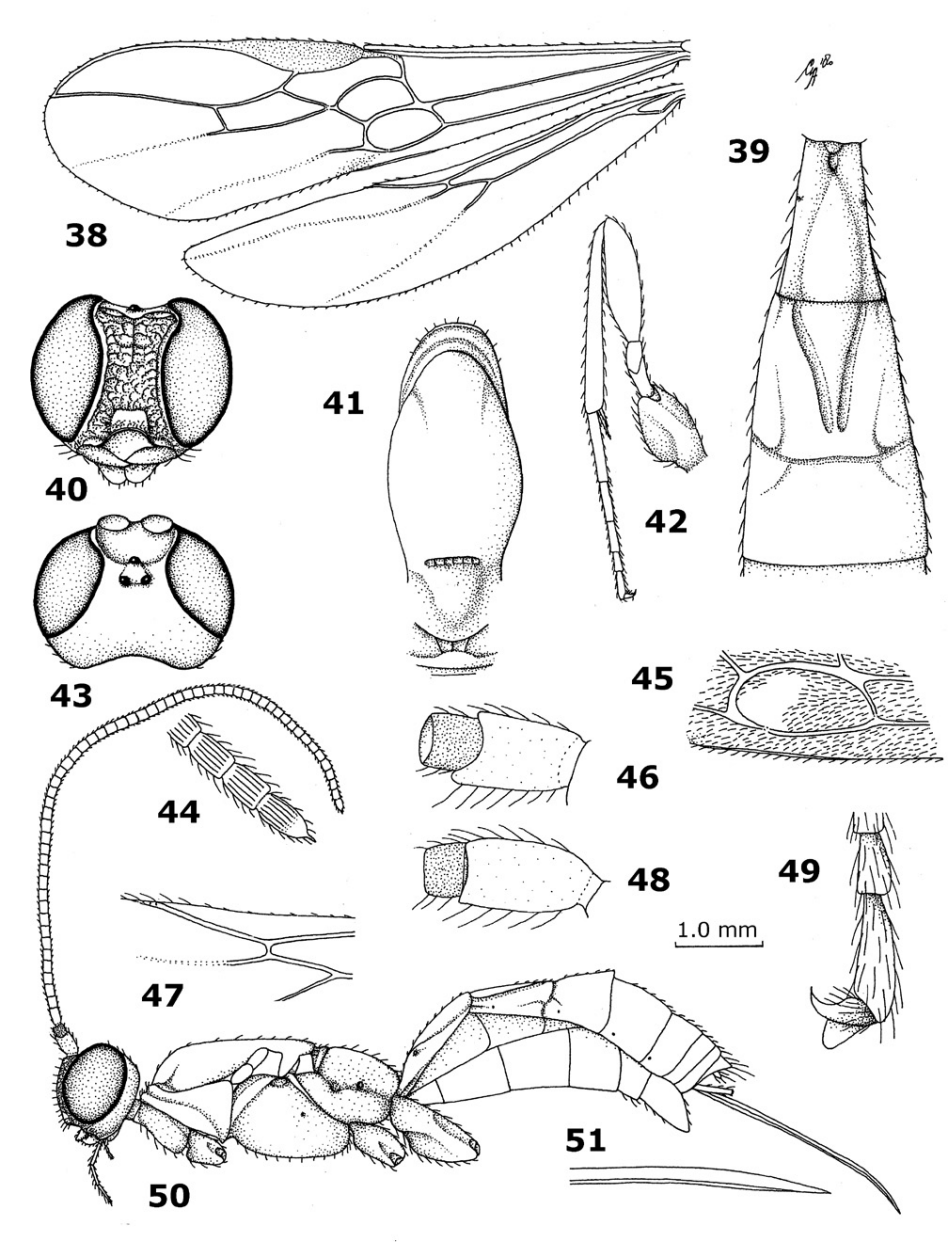
*Megalommum oculatum* Szépligeti, holotype female. **38** wings **39** first-third metasomal tergites dorsal **40** head anterior **41** mesonotum dorsal **42** hind leg **43** head dorsal **44** apex of antenna **45** first subdiscal cell of fore wing **46** scapus and pedicellus outer side **47** veins SC+R1, 2-SC+R and 1r-m of hind wing **48** scapus and pedicellus inner side **49** inner middle claw **50** habitus, lateral **51** apex of ovipositor.

**Figures 52–64.**
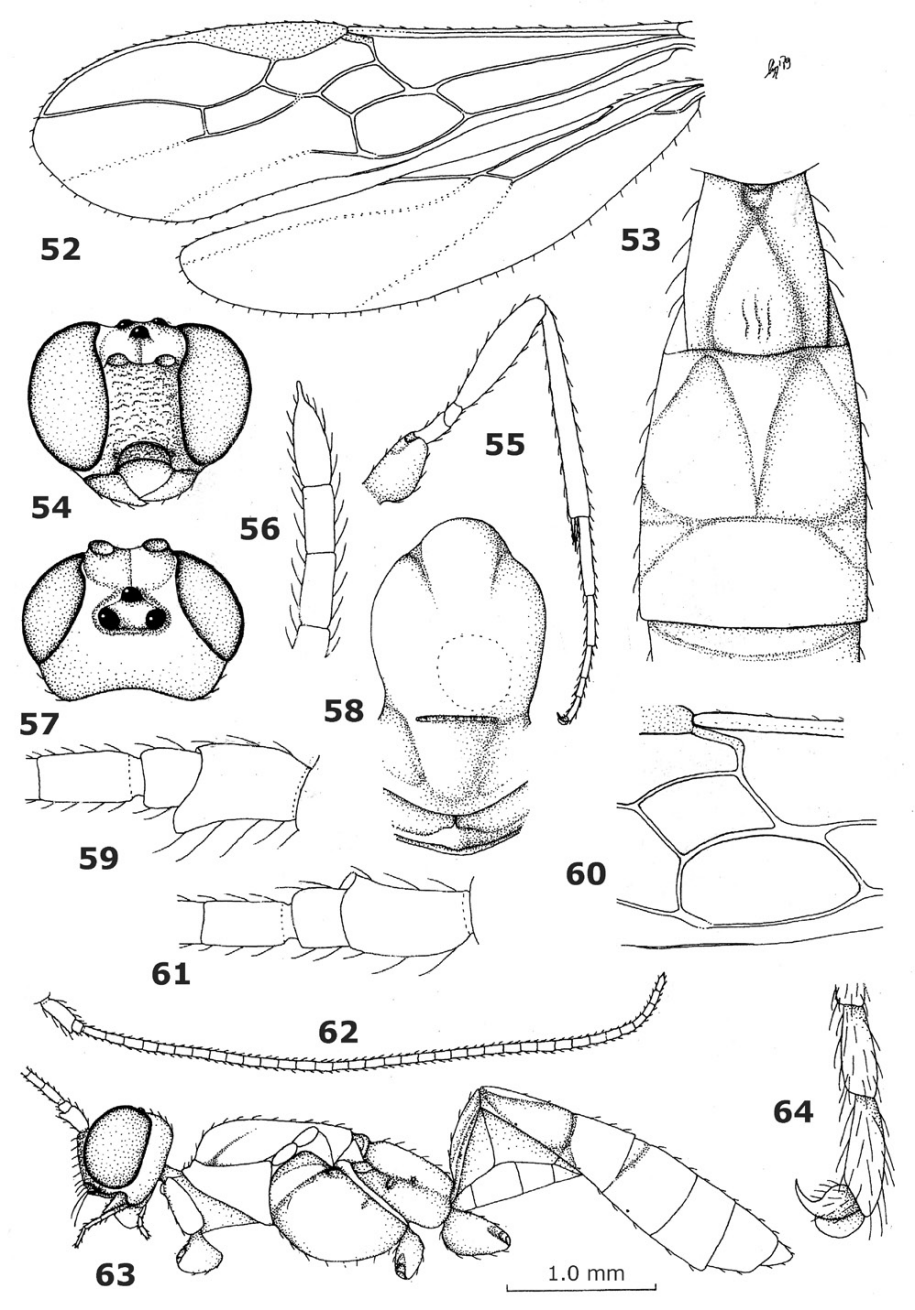
*Endovipio ceresensis* Turner, holotype male. **52** wings **53** first-third metasomal tergites dorsal **54** head anterior **55** hind leg **56** apex of antenna **57** head dorsal **58** mesonotum dorsal **59** scapus and pedicellus outer side **60** first discal and first subdiscal cells of fore wing **61** scapus and pedicellus inner side **63** habitus, lateral **64** inner hind claw.

**Figures 65–73.**
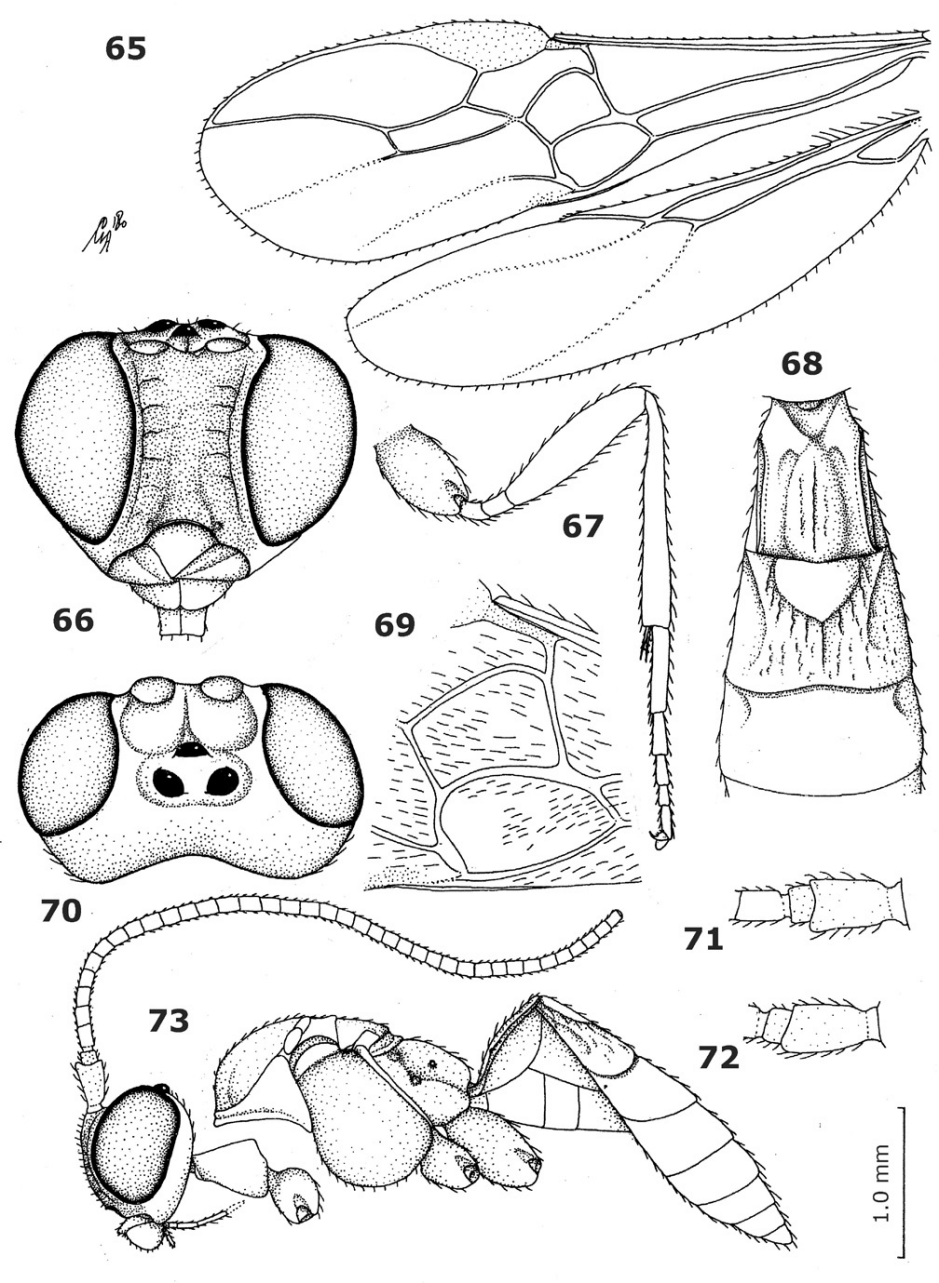
*Aphrastobracon flavipennis* Ashmead, holotype male. **65** wings **66** head anterior **67** hind leg **68** first-third metasomal tergites dorsal **69** first discal and first subdiscal cells of fore wing **70** head dorsal **71** scapus and pedicellus, outer side **72** scapus and pedicellus inner side **73** habitus lateral.

**Figures 74–76.**
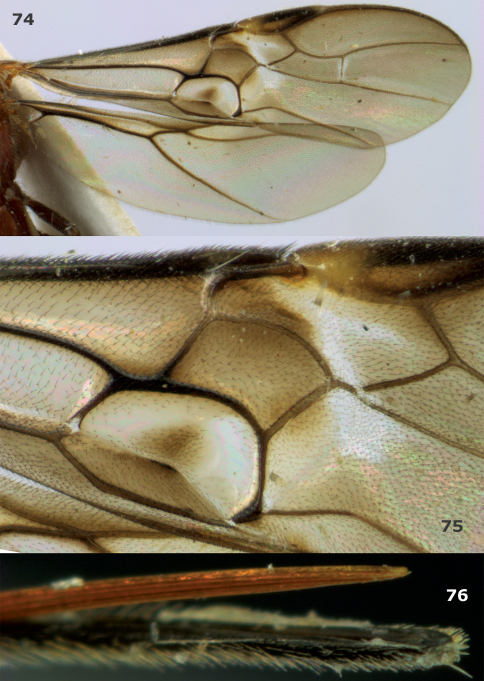
*Megalommum pistacivorae*sp. n., paratype female. **74** wings **75** detail of fore wing **76** apex of ovipositor.

**Figures 77–79.**
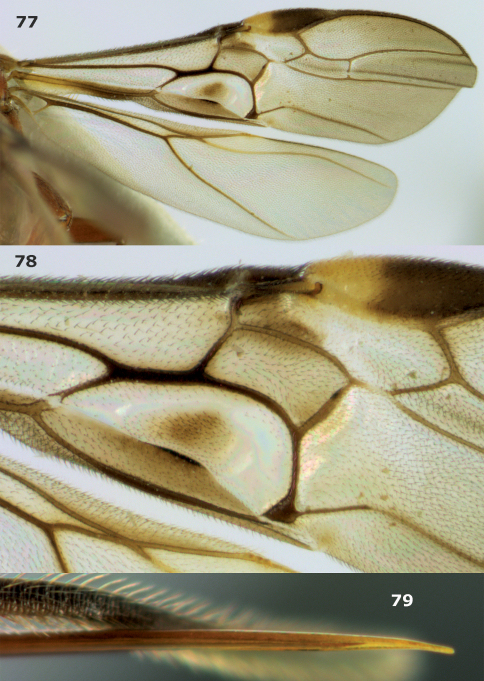
*Megalommum jacobsoni* **(**Tobias), Yemen, Al Kowd, female. **77** wings **78** detail of fore wing **79** apex of ovipositor.

## Supplementary Material

XML Treatment for Megalommum pistacivorae
